# Associations of Age, BMI, and Years of Menstruation with Proximal Femur Strength in Chinese Postmenopausal Women: A Cross-Sectional Study

**DOI:** 10.3390/ijerph13020157

**Published:** 2016-01-23

**Authors:** Huili Kang, Yu-Ming Chen, Guiyuan Han, Hua Huang, Wei-Qing Chen, Xidan Wang, Ying-Ying Zhu, Su-Mei Xiao

**Affiliations:** Department of Medical Statistics and Epidemiology, School of Public Health, Sun Yat-Sen University, Guangzhou 510080, China; kanghl3@mail2.sysu.edu.cn (H.K.); hangy3@mail2.sysu.edu.cn (G.H.); huangh237@mail2.sysu.edu.cn (H.H.); chenwq@mail.sysu.edu.cn (W.-Q.C.); wangxid@mail2.sysu.edu.cn (X.W.); missxiedou@163.com (Y.-Y.Z.)

**Keywords:** proximal femur strength, age, BMI, years of menstruation, postmenopausal women

## Abstract

This study aimed to elucidate the associations of age, BMI, and years of menstruation with proximal femur strength in Chinese postmenopausal women, which may improve the prediction of hip fracture risk. A cross-sectional study was conducted in 1322 Chinese postmenopausal women recruited from communities. DXA images were used to generate bone mineral density (BMD) and geometric parameters, including cross-sectional area (CSA), outer diameter (OD), cortical thickness (CT), section modulus (SM), buckling ratio (BR) at the narrow neck (NN), intertrochanter (IT), and femoral shaft (FS). Relationships of age, BMI, and years of menstruation with bone phenotypes were analyzed with the adjustment of height, age at menarche, total daily physical activity, education, smoking status, calcium tablet intake, *etc*. Age was associated with lower BMD, CSA, CT, SM, and higher BR (*p* < 0.05), which indicated a weaker bone strength at the proximal femur. BMI and years of menstruation had the positive relationships with proximal femur strength (*p* < 0.05). Further analyses showed that the ranges of absolute value of change slope per year, per BMI or per year of menstruation were 0.14%–1.34%, 0.20%–2.70%, and 0.16%–0.98%, respectively. These results supported that bone strength deteriorated with aging and enhanced with higher BMI and longer time of years of menstruation in Chinese postmenopausal women.

## 1. Introduction

Osteoporosis is a common skeletal disease characterized by compromised bone strength predisposing to an increased risk of fracture. Bone mineral density (BMD) is widely used in assessing bone strength and predicting the risk of fracture. Nonetheless, BMD alone is not sufficient to predict the fracture risk of an individual [1]. Studies showed that approximately half of the fracture patients had a higher BMD value than the diagnostic criteria of osteoporosis [2,3]. In this kind of patient, it may be the reduced bone geometric strength that makes the bone fragile. Studies have reported that bone geometry was one of the key determinants of bone strength and it can predict fracture risk independent of BMD [4,5,6]. Therefore, the studies on both BMD and bone geometry are indispensable for improving the accuracy of fracture risk prediction model and for the comprehensive understanding of bone strength and osteoporosis.

Age, obesity, and estrogen have substantial effects on bone. The changes of endocrine system with aging, such as the decreasing of oxidation resistance and estrogen (in women), and the increasing of endogenous glucocorticoidism levels [7], could lead to the loss of bone mass and the degradation of bone geometric strength. Obesity affects osteoporotic fractures significantly and it is ranked alongside age in importance. Studies used body mass index (BMI) as an indicator of obesity to investigate its relationship with BMD and fractures. Nonetheless, the results were inconsistent among studies [8,9,10]. Recently, Ong *et al.* found that a higher BMI was associated with a higher BMD, but this not meant a lower risk of fracture (*n* = 4288) [11]. Besides BMD, it may provide some explanation for these findings via investigating the effect of BMI on bone geometric structure. Estrogen is important for bone formation and bone growth in women [12]. A number of studies suggested that a long lifetime exposure of the endogenous estrogen may play a protective role in the development of osteoporosis and fractures [13,14].

Previous studies about the relationships between age, obesity, estrogen, and bone phenotypes, especially for the bone geometric structure, were mainly conducted in the white populations and few in Chinese. Therefore, in this study, we aimed to elucidate the associations of age, BMI and years of menstruation with proximal femur strength in Chinese postmenopausal women.

## 2. Materials and Methods 

### 2.1. Study Population

The subjects were selected from an ongoing cohort study started in 2008. It was designed to assess the environmental and genetic determinants of osteoporosis and cardiometabolic diseases [15]. Volunteers were recruited by sending invitation letters to residential buildings, by posting local advertisements, by giving health talks, and from referrals in the local community. This study used the data of the first follow-up examination performed between June 2010 and December 2013, and the data of the new participants joined in during this period. There were 1717 postmenopausal women (natural cessation of menses ≥12 months) aged 44–87 years with DXA measurements at the hip. All of them were residents of urban Guangzhou, China for more than five years. After the exclusion of subjects with chronic diseases or conditions that may affect bone and mineral metabolism: e.g., having a history of metabolic bone disorder or hip fracture, chronic medical illness, endocrine diseases including hyperthyroidism, medications that may affect bone and calcium metabolism, premature menopause age less than 40 years, major gastrointestinal operations, bilateral oophorectomy, or being prescribed drugs like bisphosphonates, selective estrogen receptor modulators, calcitonin, and active vitamin D3 metabolites, a total of 1322 subjects were included in this study. All subjects gave their informed consent for inclusion before they participated in the study. The study was conducted in accordance with the Declaration of Helsinki, and the protocol was approved by the Ethics Committee of School of Public Health of the Sun Yat-Sen University (2012.1, 5 March 2012).

### 2.2. Bone Densitometry and Hip Structure Analysis

BMD (g/cm^2^) at the left hip of all subjects was measured by using the dual-energy X-ray absorptiometry (DXA, Discovery W; Hologic Inc., Waltham, MA, USA). DXA images of the hip proximal femur were reprocessed with the Hip Structure Analysis (HSA) program included in the APEX software (v3.2, Hologic Inc., Waltham, MA, USA) by the same well trained professional. This method has been described in detail previously [16]. The HSA program automatically provides HSA indices in three regions of interest (ROIs), including: (1) narrow neck (NN), traversing the narrowest width of the femoral neck; (2) intertrochanter (IT), along the bisector of the shaft and femoral neck axes; (3) femoral shaft (FS), 2 cm distal to the midpoint of the lesser trochanter. Five HSA analyzed indices, *i.e.*, cross-sectional area (CSA, cm^2^), outer diameter (OD, cm), cortical thickness (CT, cm), section modulus (SM, cm^3^), and buckling ratio (BR), were used in this study. SM is an index of resistance to bending forces. BR describes stable configurations of thin-walled tubes subjected to compressive loads and requires an estimate of the cortical thickness. The *in vivo* precisions of BMD were 1.92%, 1.82%, and 1.40% at NN, IT, and FS, respectively.

### 2.3. Exposure and Covariate Measurements

Socio-demographic information, the history of disease and medications, reproductive histories, and lifestyle habits were collected by means of face-to-face interviews conducted by the trained staff using a structured questionnaire. Age at menarche was the age at first menses. Age at menopause was the age at the last menstrual period prior to stopping menstruation for 12 months. Years of menstruation was calculated by subtracting age at menarche from age at menopause. Total daily physical activity (metabolic equivalent, MET) was estimated by questions on the frequency and duration of physical trainings and daily activities except for sitting and lying. Smoking and alcohol drinking status were recorded. All subjects were divided into non-smokers, active smokers, or passive smokers, and non-drinkers or drinkers. Active smokers were defined as the persons who smoked more than 100 cigarettes in their lifetime. Passive smokers were those who lived or worked in the room with a person who smoked more than one cigarette or five minutes per day for at least one year. Individuals who had ever drunk alcoholic beverages at least once a week for more than six months were considered as drinkers. The active smokers and drinkers were not included in the statistical analyses, as there were only six (0.4%) active smokers and 42 (3.1%) drinkers in this sample. Calcium tablet intake was defined as taking calcium supplements more than 30 times over the past year. The height and weight were measured to the nearest 0.1 cm and 0.1 kg, respectively, without shoes and in light clothing. BMI was calculated as weight (kg) divided by height (m) squared.

### 2.4. Statistical Methods

Continuous or categorical variables were expressed as the mean and standard deviation (SD) or number (percentage), respectively. The associations between age, BMI, years of menstruation, BMD, and HSA indices were detected using the linear regression model with the adjustment of the confounding factors. Age specific values of bone phenotypes were obtained in each five year age group: ≤55, 56–60, 61–65, and ≥66 years old. BMI specific values of bone phenotypes were evaluated in four subgroups: underweight (<18.5 kg/m^2^), normal weight (18.5–23.9 kg/m^2^), overweight (24.0–27.9 kg/m^2^), and obese (≥28.0 kg/m^2^), classified according to the Working Group on Obesity in China (WGOC). The years of menstruation specific values were also obtained in four subgroups: ≤31, 32–36, 37–41, and ≥42 years. Multivariate analysis of covariance (ANCOVA) was used to compare the differences of bone phenotypes among different age, BMI, and years of menstruation groups. Least-significant difference (LSD) test was used for the comparisons between groups. In this study, the confounding effects of height, age at menarche, total daily physical activity, education, smoking status, and calcium tablet intake were adjusted in all of the statistical analyses. In addition, when age, BMI or years of menstruation was analyzed, two of the variables, *i.e.*, age, weight, and years of menstruation, were also adjusted as covariates in the analysis. Slope of change of each bone phenotype was calculated by a partial regression coefficient obtained from the multiple linear regression analysis in all subjects divided by the overall mean. The statistical analyses were performed using SPSS (v17.0, SPSS Inc., Chicago, IL, USA). A two-sided *p* < 0.05 was considered as significance.

## 3. Results

### 3.1. General Characteristics of the Subjects

The basic information of the 1322 participants is shown in Table 1. The mean (SD) of age, BMI, years of menstruation, age at menarche, and total daily physical activity was 59.6 (5.0) years, 23.4 (3.3) kg/m^2^, 36.3 (3.4) years, 14.1 (1.7) years, and 17.0 (6.5) MET·h/d, respectively. In these subjects, there were around 26.8% passive smokers. Approximately 35.0% persons were taking calcium tablets more than 30 times over the past year.

**Table 1:** Basic information of studied subjects (*n* = 1322).

Variable	Mean/*n*	SD/Percentage
Age (year)	59.6	5.0
Height (cm)	154.9	5.4
Weight (kg)	56.0	8.4
BMI (kg/m^2^)	23.4	3.3
Years of menstruation (year)	36.3	3.4
Age at menarche	14.1	1.7
Total daily physical activity (MET·h/d)	17.0	6.5
Education		
primary school or below	105	7.9%
junior high school	315	23.8%
senior high school	654	49.5%
college degree or above	248	18.8%
Smoking status		
passive smokers	354	26.8%
no	968	73.2%
Calcium tablet intake		
yes	463	35.0%
no	859	65.0%

Data were expressed as mean and SD or n and percentage.

### 3.2. Associations of Age, BMI, and Years of Menstruation with BMD and HSA Indices

Table 2 displays the results of associations between age, BMI, years of menstruation, and bone phenotypes with the adjustment of height, age at menarche, total daily physical activity, education, smoking status, calcium tablet intake, *etc.* Age, BMI, and years of menstruation were significantly associated with most of the studied bone parameters in all of the three ROI regions (*p* < 0.05). Age had negative relationships with BMD, CSA, and CT, and showed a positive relationship with BR (*p* < 0.05). For BMI and years of menstruation, they were positively related to BMD, CSA, and CT and negatively related to BR (*p* < 0.05). The total variances of BMD, CSA, CT, and BR explained by these three variables were approximately 23.1%, 29.9%, 22.4%, and 12.7% at NN, 22.0%, 27.2%, 21.3%, and 16.1% at IT, 19.6%, 29.1%, 16.6%, and 10.2% at FS, respectively. Age was negatively associated, and BMI and years of menstruation positively associated, with SM at the NN and IT sites as well (*p* < 0.05) and together they explained around 16.1% and 24.7% of the variances of SM at NN and IT, respectively. For SM at FS, there were positive relationships with BMI and years of menstruation(*p* < 0.05), but not with age (*p* > 0.05). OD was positively associated with age and BMI at all three sites and inversely with years of menstruation at NN (*p* < 0.05). For the total variation explained by these three variables, BMI was the greatest contributor with the explained variance about 10%–20% for most bone phenotypes, followed by age contributing to about 3%–10% of the variation. The contribution of years of menstruation was from 0.3% to 2.3%.

**Table 2:** Association results of age, BMI and years of menstruation with BMD and HSA indices.

ROI Site	Variable	Cumulative Variability Explained	Age			BMI			Years of Menstruation		
			*β*	*p*	Variability Explained by Variable	*β*	*p*	Variability Explained by Variable	*β*	*p*	Variability Explained by Variable
NN	BMD	23.1%	−0.008	<0.001	8.4%	0.014	<0.001	13.0%	0.005	<0.001	1.7%
	CSA	29.9%	−0.018	<0.001	7.9%	0.046	<0.001	20.7%	0.012	<0.001	1.3%
	OD	2.1%	0.005	<0.001	1.2%	0.006	0.003	0.7%	−0.005	0.026	0.3%
	CT	22.4%	−0.002	<0.001	8.3%	0.003	<0.001	12.4%	0.001	<0.001	1.7%
	SM	16.1%	−0.007	<0.001	3.3%	0.020	<0.001	12.0%	0.005	0.002	0.7%
	BR	12.7%	0.149	<0.001	6.8%	−0.177	<0.001	4.6%	−0.101	<0.001	1.3%
IT	BMD	22.0%	−0.007	<0.001	6.9%	0.015	<0.001	13.0%	0.006	<0.001	2.0%
	CSA	27.2%	−0.031	<0.001	5.6%	0.092	<0.001	19.6%	0.029	<0.001	1.9%
	OD	4.4%	0.008	<0.001	1.1%	0.020	<0.001	3.3%	−0.002	0.572	0.0%
	CT	21.3%	−0.004	<0.001	7.0%	0.007	<0.001	12.4%	0.003	<0.001	1.9%
	SM	24.7%	−0.018	<0.001	2.1%	0.094	<0.001	21.3%	0.023	<0.001	1.3%
	BR	16.1%	0.096	<0.001	7.1%	-0.136	<0.001	6.7%	−0.085	<0.001	2.3%
FS	BMD	19.6%	−0.008	<0.001	4.3%	0.021	<0.001	13.9%	0.007	<0.001	1.4%
	CSA	29.1%	−0.015	<0.001	3.1%	0.069	<0.001	24.5%	0.016	<0.001	1.5%
	OD	5.0%	0.004	<0.001	1.4%	0.010	<0.001	3.5%	−0.002	0.148	0.2%
	CT	16.6%	−0.004	<0.001	4.2%	0.010	<0.001	11.2%	0.003	<0.001	1.1%
	SM	27.2%	0.000	0.777	0.0%	0.040	<0.001	26.7%	0.005	0.012	0.5%
	BR	10.2%	0.028	<0.001	3.5%	−0.052	<0.001	5.3%	−0.028	<0.001	1.4%

The associations between age, BMI, years of menstruation, BMD, and HSA indices were detected using the linear regression model with the adjustment of the confounding factors, *i.e.*, height, age at menarche, total daily physical activity, education, smoking status, and calcium tablet intake. *β*, partial regression coefficient; ROI, regions of interest; NN, narrow neck; IT, intertrochanter; FS, femoral shaft; BMD, bone mineral density; CSA, cross-sectional area; OD, outer diameter; CT, cortical thickness; SM, section modulus; BR, buckling ratio.

### 3.3. Age Specific Values of BMD and HSA Indices

**Figure 1:**
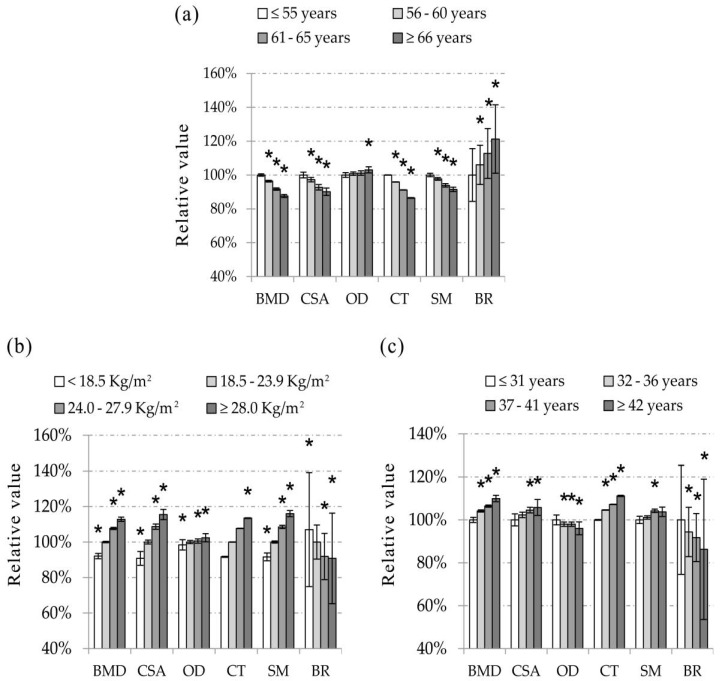
The relative values of bone mineral density (BMD) and Hip Structure Analysis (HSA) indices of each subgroup of age (**a**); BMI (**b**) or years of menstruation (**c**) to the reference group at narrow neck (NN). The values were expressed as percent relative to the group of age ≤55 years, BMI = 18.5–23.9 kg/m^2^ and years of menstruation ≤31 years, respectively. Values were adjusted for height, age at menarche, total daily physical activity, education, smoking status and calcium tablet intake. In addition, when age, BMI or years of menstruation was analyzed, two of the variables, *i.e.*, age, weight and years of menstruation, were also adjusted as covariates in the analysis. Vertical bars represent the standard errors of the mean value. * *p* < 0.05 *vs.* the reference group; NN, narrow neck; BMD, bone mineral density; CSA, cross-sectional area; OD, outer diameter; CT, cortical thickness; SM, section modulus; BR, buckling ratio.

Table 3 shows the mean values of BMD and HSA indices in each age groups after the adjustment of weight, years of menstruation, height, age at menarche, total daily physical activity, education, smoking status, and calcium tablet intake. The mean differences of bone phenotypes between the reference group of age ≤55 years and the group of age = 56–60, 61–65 or ≥65 years are shown in supplemental table S1. Figure 1 (a, for NN) and supplemental Figure S1 (a, for IT and FS) illustrates the relative values of bone phenotype of each subgroup to the age ≤55 years group. As shown in Table 3, Figure 1a and supplemental Figure S1a, BMD, CSA, CT, SM were lower and OD, BR were higher in the older age group (all *p* < 0.01), except that there was no significant difference for SM at FS among the age groups (*p* > 0.05). Table 3 also shows a slope of each bone phenotype per year of age. In each skeletal region, the absolute slope value of CSA, CT, SM, and BR were similar to that of BMD except for SM at FS. The ranges of absolute value of slope per year were 0.43%–1.34% for BMD, CSA, CT, BR, and 0.14%–0.16% for OD at three ROI regions, and 0.52%–0.64% for SM at NN and IT, respectively. The slope of BR was steeper than all of the others indices in the same region.

**Table 3:** BMD and HSA indices of subjects in different age groups.

ROI Site	Variable	Age (year)				*p*	Slope/Year
		≤55 (*n* = 300)	56–60 (*n* = 519)	61–65 (*n* = 327)	≥66 (*n* = 176)		
NN	BMD (g/cm^2^)	0.879 (0.007)	0.847 (0.005) **^a^**	0.806 (0.007)) **^a^**	0.770 (0.009) **^a^**	<0.001	−0.96%
	CSA (cm^2^)	2.508 (0.017)	2.439 (0.013) **^a^**	2.326 (0.016) **^a^**	2.260 (0.022) **^a^**	<0.001	−0.75%
	OD (cm)	3.013 (0.014)	3.038 (0.010)	3.048 (0.013)	3.105 (0.018) **^a^**	0.001	0.16%
	CT (cm)	0.170 (0.001)	0.163 (0.001) **^a^**	0.155 (0.001) **^a^**	0.147(0.002) **^a^**	<0.001	−1.24%
	SM (cm^3^)	1.131 (0.010)	1.105 (0.008) **^a^**	1.062 (0.010) **^a^**	1.035 (0.013) **^a^**	<0.001	−0.64%
	BR	10.235 (0.156)	10.849 (0.116) **^a^**	11.538 (0.147) **^a^**	12.411 (0.202) **^a^**	<0.001	1.34%
IT	BMD (g/cm^2^)	0.903 (0.008)	0.858 (0.006) **^a^**	0.823 (0.007) **^a^**	0.794 (0.010) **^a^**	<0.001	−0.82%
	CSA (cm^2^)	4.481 (0.036)	4.301(0.027) **^a^**	4.153 (0.034) **^a^**	4.020 (0.047) **^a^**	<0.001	−0.73%
	OD (cm)	5.214 (0.021)	5.279 (0.016) **^a^**	5.295 (0.020) **^a^**	5.346 (0.028) **^a^**	0.002	0.15%
	CT (cm)	0.394 (0.004)	0.376 (0.003) **^a^**	0.359 (0.003) **^a^**	0.343 (0.005) **^a^**	0.001	−1.08%
	SM (cm^3^)	3.631 (0.035)	3.492 (0.026) **^a^**	3.421 (0.033) **^a^**	3.352 (0.045) **^a^**	<0.001	−0.52%
	BR	8.057 (0.099)	8.583 (0.073) **^a^**	8.927(0.093) **^a^**	9.470 (0.127) **^a^**	<0.001	1.11%
FS	BMD (g/cm^2^)	1.370 (0.010)	1.344 (0.008) **^a^**	1.314 (0.010) **^a^**	1.258 (0.013) **^a^**	<0.001	−0.60%
	CSA (cm^2^)	3.581 (0.023)	3.524 (0.017) **^a^**	3.474 (0.022) **^a^**	3.351 (0.030) **^a^**	<0.001	−0.43%
	OD (cm)	2.751 (0.010)	2.759 (0.008)	2.783 (0.010) **^a^**	2.804 (0.013) **^a^**	0.006	0.14%
	CT (cm)	0.517 (0.005)	0.506 (0.004)	0.488 (0.005) **^a^**	0.461 (0.007) **^a^**	<0.001	−0.80%
	SM (cm^3^)	1.841 (0.013)	1.837 (0.010)	1.853 (0.012)	1.832 (0.017)	0.681	0.00%
	BR	2.863 (0.042)	2.965 (0.031)	3.043 (0.040) **^a^**	3.278 (0.054) **^a^**	<0.001	0.93%

Values were presented as mean (SE). Bone mineral density (BMD) and Hip Structural Analysis (HSA) indices at three regions of interest (ROI) were adjusted by weight, years of menstruation, height, age at menarche, total daily physical activity, education, smoking status, and calcium tablet intake. *p* values were for the differences among subgroups analyzed using ANCOVA. Slope/year was the partial regression coefficient of each bone phenotype from all subjects in percent of the overall mean on age adjusted for the above covariates. **^a^** *p* < 0.05 *vs.* the group of age ≤55 years; ROI, regions of interest; NN, narrow neck; IT, intertrochanter; FS, femoral shaft; BMD, bone mineral density; CSA, cross-sectional area; OD, outer diameter; CT, cortical thickness; SM, section modulus; BR, buckling ratio.

### 3.4. BMI Specific Values of BMD and HSA Indices

The mean values of BMD and HSA indices in each BMI group after adjusting for age, years of menstruation, height, age at menarche, total daily physical activity, education, smoking status, and calcium tablet intake are displayed in Table 4. The subjects were divided into four subgroups in this study, *i.e.*, underweight (<18.5 kg/m^2^), normal weight (18.5–23.9 kg/m^2^), overweight (24.0–27.9 kg/m^2^), and obese (≥28.0 kg/m^2^). The mean differences of bone phenotype values between the normal weight group and the other subgroups are displayed in supplemental Table S2. The relative values of bone phenotypes for each group in percent based on the values of normal weight group were calculated and illustrated in Figure 1 (b, for NN) and supplemental Figure S1 (b, for IT and FS). Data showed that there were significant differences among BMI related subgroups for all of the studied bone phenotypes (*p* < 0.01, Table 4). In the group with the larger value of BMI, the values of BMD, CSA, OD, CT, and SM were larger and BR was smaller at all ROI sites (Figure 1b) and supplemental Figure S1b. The slopes of CSA, CT, SM, and BR were as steep as that of BMD in each ROI region. The absolute values of slope per unit BMI varied from 1.57% to 2.70% for BMD, CSA, CT, SM, and BR, and were less than 0.38% for OD (Table 4), respectively.

### 3.5. Years of Menstruation Specific Values of BMD and HSA Indices 

The years of menstruation specific mean values of BMD and HSA indices after the adjustment of age, weight, height, age at menarche, total daily physical activity, education, smoking status and calcium tablet intake are showed in Table 5. The mean differences of bone phenotypes between the group with years of menstruation ≤31 years and the other subgroups are shown in supplemental Table S3. The relative values of bone phenotypes for each subgroup in percent classified by years of menstruation are illustrated in Figure 1 (c, for NN) and supplemental Figure S1 (c, for IT and FS), by using the lowest exposure group (≤31 years of menstruation) as the reference group. As shown in Table 5, Figure 1c and supplemental Figure S1c, BMD, CSA, CT, and SM were higher and BR was lower with a longer time of years of menstruation at all ROI sites (*p* < 0.05). The difference of OD at NN was significant among subgroups of years of menstruation (*p* < 0.05), whereas no difference was observed for it at IT and FS (*p* > 0.05). The range of absolute values of slope per year of menstruation was 0.16%–0.98% for the significant bone phenotypes at the three ROI sites.

**Table 4:** BMD and HSA indices of subjects in different BMI groups.

ROI Site	Variable	BMI (kg/m^2^)				*p*	Slope/BMI
		<18.5 (*n* = 67)	18.5–23.9 (*n* = 743)	24.0–27.9 (*n* = 405)	≥28.0 (*n* = 107)		
NN	BMD (g/cm^2^)	0.746 (0.015) **^a^**	0.810 (0.004)	0.872 (0.006) **^a^**	0.914 (0.012) **^a^**	<0.001	1.68%
	CSA (cm^2^)	2.109 (0.039) **^a^**	2.323 (0.011)	2.524 (0.015) **^a^**	2.683 (0.029) **^a^**	<0.001	1.91%
	OD (cm)	2.987 (0.029)	3.035 (0.009)	3.052 (0.012)	3.109 (0.023) **^a^**	0.004	0.20%
	CT (cm)	0.143 (0.003) **^a^**	0.156 (0.001)	0.168 (0.001) **^a^**	0.177 (0.002) **^a^**	<0.001	1.86%
	SM (cm^3^)	0.966 (0.022) **^a^**	1.054 (0.006)	1.144 (0.009) **^a^**	1.223 (0.017) **^a^**	<0.001	1.83%
	BR	12.216 (0.321) **^a^**	11.414 (0.096)	10.486 (0.130) **^a^**	10.363 (0.255) **^a^**	<0.001	−1.60%
IT	BMD (g/cm^2^)	0.746 (0.016) **^a^**	0.826 (0.005)	0.891 (0.006) **^a^**	0.944 (0.013) **^a^**	<0.001	1.76%
	CSA (cm^2^)	3.647 (0.076) **^a^**	4.114 (0.023)	4.508 (0.031) **^a^**	4.813 (0.060) **^a^**	<0.001	2.16%
	OD (cm)	5.074 (0.044) **^a^**	5.251 (0.013)	5.337 (0.018) **^a^**	5.356 (0.035) **^a^**	<0.001	0.38%
	CT (cm)	0.324 (0.008) **^a^**	0.360 (0.002)	0.389 (0.003) **^a^**	0.414 (0.006) **^a^**	<0.001	1.89%
	SM (cm^3^)	2.780 (0.074) **^a^**	3.331 (0.022)	3.752 (0.030) **^a^**	4.009 (0.058) **^a^**	<0.001	2.70%
	BR	9.688 (0.203) **^a^**	8.890 (0.061)	8.267 (0.082) **^a^**	7.989 (0.161) **^a^**	<0.001	−1.57%
FS	BMD (g/cm^2^)	1.165 (0.021) **^a^**	1.300 (0.006)	1.383 (0.009) **^a^**	1.455 (0.017) **^a^**	<0.001	1.58%
	CSA (cm^2^)	2.979 (0.049) **^a^**	3.397 (0.015)	3.672 (0.020) **^a^**	3.908 (0.039) **^a^**	<0.001	1.97%
	OD (cm)	2.703 (0.021) **^a^**	2.749 (0.006)	2.800 (0.009) **^a^**	2.831 (0.017) **^a^**	<0.001	0.36%
	CT (cm)	0.425 (0.011) **^a^**	0.483 (0.003)	0.522 (0.004) **^a^**	0.556 (0.009) **^a^**	<0.001	2.01%
	SM (cm^3^)	1.539 (0.027) **^a^**	1.780 (0.008)	1.942 (0.011) **^a^**	2.077 (0.022) **^a^**	<0.001	2.17%
	BR	3.455 (0.087) **^a^**	3.065 (0.026)	2.887 (0.035) **^a^**	2.727 (0.069) **^a^**	<0.001	−1.73%

Values were presented as mean (SE). Bone mineral density (BMD) and Hip Structural Analysis (HSA) indices at three regions of interest (ROI) were adjusted by age, years of menstruation, height, age at menarche, total daily physical activity, education, smoking status and calcium tablet intake. *p* values were for the differences among subgroups analyzed using ANCOVA. Slope/BMI was the partial regression coefficient of each bone phenotype from all subjects in percent of the overall mean on BMI adjusted for the above covariates. **^a^** *p* < 0.05 *vs.* the group of BMI = 18.5–23.9 kg/m^2^; ROI, regions of interest; NN, narrow neck; IT, intertrochanter; FS, femoral shaft; BMD, bone mineral density; CSA, cross-sectional area; OD, outer diameter; CT, cortical thickness; SM, section modulus; BR, buckling ratio.

**Table 5:** BMD and HSA indices of subjects in different years of menstruation groups.

ROI Site	Variable	Years of Menstruation (year)				*p*	Slope/Year of Menstruation
		≤31 (*n* = 112)	32–36 (*n* = 556)	37–41 (*n* = 586)	≥42 (*n* = 68)		
NN	BMD (g/cm^2^)	0.793 (0.012)	0.826 (0.005) **^a^**	0.844 (0.005) **^a^**	0.872 (0.015) **^a^**	<0.001	0.60%
	CSA (cm^2^)	2.326 (0.028)	2.380 (0.013)	2.432 (0.013) **^a^**	2.461 (0.037) **^a^**	0.001	0.50%
	OD (cm)	3.104 (0.023)	3.042 (0.010) **^a^**	3.042 (0.010) **^a^**	2.982 (0.030) **^a^**	0.011	−0.16%
	CT (cm)	0.152 (0.002)	0.159 (0.001) **^a^**	0.163 (0.001) **^a^**	0.169 (0.003) **^a^**	<0.001	0.62%
	SM (cm^3^)	1.064 (0.017)	1.076 (0.008)	1.109 (0.008) **^a^**	1.104 (0.022)	0.012	0.46%
	BR	11.887 (0.254)	11.218 (0.115) **^a^**	10.909 (0.112) **^a^**	10.255 (0.327) **^a^**	<0.001	−0.91%
IT	BMD (g/cm^2^)	0.799 (0.012)	0.841 (0.006) **^a^**	0.866 (0.006) **^a^**	0.895 (0.016) **^a^**	<0.001	0.71%
	CSA (cm^2^)	4.052 (0.059)	4.211 (0.027) **^a^**	4.343 (0.026) **^a^**	4.441 (0.076) **^a^**	<0.001	0.68%
	OD (cm)	5.325 (0.035)	5.265 (0.016) **^a^**	5.283 (0.015) **^a^**	5.248 (0.045) **^a^**	0.351	−0.04%
	CT (cm)	0.349 (0.006)	0.366 (0.003) **^a^**	0.379 (0.003) **^a^**	0.389 (0.008) **^a^**	<0.001	0.81%
	SM (cm^3^)	3.316 (0.057)	3.445 (0.026) **^a^**	3.543 (0.025) **^a^**	3.636 (0.074) **^a^**	0.001	0.66%
	BR	9.332 (0.160)	8.827 (0.072) **^a^**	8.448 (0.071) **^a^**	8.141 (0.206) **^a^**	<0.001	−0.98%
FS	BMD (g/cm^2^)	1.282 (0.017)	1.313 (0.008)	1.351 (0.007) **^a^**	1.382 (0.021) **^a^**	<0.001	0.53%
	CSA (cm^2^)	3.393 (0.038)	3.457 (0.017)	3.550 (0.017) **^a^**	3.627 (0.049) **^a^**	<0.001	0.46%
	OD (cm)	2.794 (0.017)	2.774 (0.008)	2.761 (0.008)	2.757 (0.022)	0.316	−0.07%
	CT (cm)	0.475 (0.009)	0.490 (0.004)	0.507 (0.004) **^a^**	0.521 (0.011) **^a^**	<0.001	0.60%
	SM (cm^3^)	1.821 (0.021)	1.823 (0.010)	1.855 (0.009)	1.903 (0.027) **^a^**	0.018	0.27%
	BR	3.204 (0.068)	3.078 (0.031)	2.914 (0.030) **^a^**	2.828 (0.088) **^a^**	<0.001	−0.93%

Values were presented as mean (SE). Bone mineral density (BMD) and Hip Structural Analysis (HSA) indices at three regions of interest (ROI) were adjusted by age, weight, height, age at menarche, total daily physical activity, education, smoking status, and calcium tablet intake. *p* values were for the differences among subgroups analyzed using ANCOVA. Slope/year of menstruation was the partial regression coefficient of each bone phenotype from all subjects in percent of the overall mean on years of menstruation adjusted for the above covariates. **^a^** *p* < 0.05 *vs.* the group of years of menstruation ≤31 years; ROI, regions of interest; NN, narrow neck; IT, intertrochanter; FS, femoral shaft; BMD, bone mineral density; CSA, cross-sectional area; OD, outer diameter; CT, cortical thickness; SM, section modulus; BR, buckling ratio.

## 4. Discussion

In this study, we investigated the relationships of proximal femur strength with age, BMI, and years of menstruation in postmenopausal women of south China. The results showed that age was negatively and BMI and years of menstruation were positively associated with BMD and proximal femur strength. 

Evidences from this study supported that the bone becomes more fragile with the increase of age. Data showed that BMD, CSA, CT, and SM were lower and OD, BR were higher in the older age group. These results were consistent with previous studies [17,18]. Narrowing CSA accompanied with thinning CT may contribute to smaller mechanical strength and lower bending stresses along the cortical surfaces of bone. The speed of endocortical bone resorption exceeds the periosteal bone apposition with aging, which may be the reason leading to the thinning of CT [19]. The decline of SM, an index of the strength resistance to bending and torsional stresses, and the increment of BR which is an estimate of cortical stability in buckling, revealed that the bone becomes weaker, more unstable, and more inclined to fracture with aging. The deterioration of bone with aging may be attributed to the age related degradation and changes, such as the reduced antioxidant capacity, sex hormone deficiency, glucocorticoid excess, and decreased physical activity [7].

BMI plays an important role in the determination of hip BMD and bone geometry. The results displayed that BMD, CSA, CT, SM, and OD were positively correlated, and BR was negatively correlated, with BMI. It may imply that a higher BMI could bring a stronger hip bone strength. This finding was in accordance with the study conducted in 4642 postmenopausal non-Hispanic whites women aged 50–79 years. Their results showed that the femur BMD, CSA, and SM were larger, and femur BR was smaller with a higher BMI [20]. Studies performed in adolescents also found that the obese and overweight groups had significantly higher CSA and SM in comparison to the normal weight group [21,22]. These associations are probably attributed to the relatively higher lean mass and fat mass in the higher BMI individuals. Lean mass may improve bone strength via the mechanical stresses which could stimulate the beneficial response of bone [23]. Fat mass can directly influence bone through gravitational loading. In addition, adipose tissue may exert positive effects on bone strength indirectly via enhancing estrogen production [24]. Whereas, some investigations reported that fat mass was inversely associated with BMD [25,26] and bone geometric strength [27]. The adverse impact of fat mass on bone could be due to the induced inflammatory cytokines [28], increased parathyroid hormone concentration [29], and the commitment of mesenchymal stem cells into adipogenic and osteoblastogenic cells [24]. Further research is desired to investigate the influence of lean mass and fat mass on bone strength.

Our data suggested that a longer time of years of menstruation led to a more stable bone geometric structure and a stronger bone strength. BMD, CSA, CT, and SM had positive association, and BR had a negative association, with years of menstruation. Several studies had reported the association of years of menstruation with BMD [13,30,31,32]. Most of them suggested that the long lifetime cumulative exposure to estrogens was protective against osteoporosis. Nguyen *et al.* [30] found that BMD at the lumbar spine and femoral neck increased by 2%–3% for every 10 years increase in years of estrogen exposure in 1,091 Dubbo women aged 70.0 (7.2) years. In our study, BMD at the three ROI sites increased by 0.53%–0.71% per year of menstruation. The resulting variations between the two studies could be caused by the differentiations of measured regions, adjusted covariates, and studied populations, *i.e.*, the ethnicity is different and the population of this study is younger with the age being of 59.6 (5.0) years. It is known that the rate of bone loss was fast during the first decade years after menopause [33]. The underlying mechanism of the effect of estrogen deficiency on the skeleton could be summarized as follows. The loss of estrogen may increase the rate of bone remodeling via the upregulation of the formation of osteoclasts and osteoblasts in the marrow [34], but the lifespan of osteoclasts are extended and the lifespan of osteoblasts are shortened which could result in the imbalance of bone resorption and formation [35]. Moreover, the deficiency of estrogen increases the sensitivity of bone to parathyroid hormone [33] and this further enhances the resorption. Estrogen deficiency also has a negative effect on the extraskeletal calcium homeostasis [36]. All these could give rise to the decrease of bone mass and have an adverse impact on the bone structure.

In this study, age, BMI, and years of menstruation were significantly associated with proximal femur strength. The total proportion of the variance explained by the three studied variables was around 20% for most of the bone phenotypes, *i.e.*, BMD, CSA, CT, SM, and BR. Therein, BMI was the biggest contributor and explained around 10%–20% of the variances of most indices; followed by age, which explained about 3%–10% of the variation. These results indicate that weight-bearing of a heavier load and age-related changes could have vital effects on bone. Years of menstruation explained 0.3%–2.3% of the variances of bone phenotypes, which was similar to a study conducted in the white postmenopausal women aged 60–86 years [37]. Their results showed that the reproductive years explained about 2.43% of the variance of total hip BMD. Compared with age and BMI, the effects of duration of exposure to estrogen expressed as the years of menstruation were smaller on bone phenotypes. These findings imply that it is meaningful to pay more attention to the changes along with age growth and keep an appropriate body weight to maintain the strength and health of bones.

The findings from this study could have a good generalization for ordinary urban postmenopausal women in southern China, as the results were obtained from a considerable sample size with 1322 subjects recruited from the communities in Guangzhou city. To the best of our knowledge, this is the first study to investigate the effect of years of menstruation on bone geometry in Chinese postmenopausal women with a more comprehensive considering of the consequences of long-term exposure of estrogen on bone. Nonetheless, this study may have some limitations. Firstly, the accuracy of describing the bones’ three-dimensional geometric features is restricted by the inherent limitations of DXA technology. Whereas, researches have testified that the geometric features described by using two-dimensional data derived from DXA were highly correlated with a true three-dimensional method [38]. Secondly, this study had no information about the reproductive factors, *i.e.*, the pregnancy, duration of lactation, and use of oral contraceptive, which could occur in the long period between menarche and menopause and could affect the level of estrogen. Whereas, the years of menstruation is a reasonable approximation of the endogenous estrogen exposure. Studies found that it was not necessary to use more reproductive factors besides age at menopause and menarche to determine the total duration of endogenous estrogen exposure [31]. Years of menstruation may also be the most appropriate alternative at present because of its simple, feasible, and low-cost obtained way. Thirdly, this study had no detailed information about the dose of calcium intake despite giving full consideration to the effects of major confounding factors, *i.e.*, weight, height, age at menarche, total daily physical activity, education, smoking status, and calcium tablet intake. Finally, the study design was cross-sectional; therefore, the findings cannot establish the temporal relationships or causality. Longitudinal analysis of their effects on bone phenotypes will be of interest when the ongoing cohort study has been completed.

## 5. Conclusions

The present study supported that age, BMI, and years of menstruation were vital determinants of proximal femur strength in Chinese postmenopausal women, which deteriorated with aging, and enhanced with higher BMI and longer time of years of menstruation. These findings deepened our understanding of the impacts of aging, weight, and exposure of estrogen on bone, and provided information for the maintainence of bone strength and health in Chinese postmenopausal women.

## Supplementary Materials

The following are available online at www.mdpi.com/www.mdpi.com/1660-4601/13/2/157/s1, Figure S1: The relative values of bone mineral density (BMD) and Hip Structure Analysis (HSA) indices of each subgroup of age (**a**); BMI (**b**) or years of menstruation (**c**) to the reference group at intertrochanter (IT) and femoral shaft (FS) regions. The values were expressed as percent relative to the group of age ≤55 years, BMI = 18.5–23.9 kg/m^2^ and years of menstruation ≤31 years, respectively. Values were adjusted for height, age at menarche, total daily physical activity, education, smoking status, calcium tablet intake. In addition, when age, BMI or years of menstruation was analyzed, two of the variables, *i.e.*, age, weight and years of menstruation, were also adjusted as covariates in the analysis. Vertical bars represent the standard errors of the mean value. ***** *p* < 0.05 *vs.* the reference group; IT, intertrochanter; FS, femoral shaft; BMD, bone mineral density; CSA, cross-sectional area; OD, outer diameter; CT, cortical thickness; SM, section modulus; BR, buckling ratio., Table S1: Mean differences of BMD and HSA indices between the age group of ≤55 years and the group of 56–60, 61–65, or ≥65 years, Table S2: Mean differences of BMD and HSA indices between the BMI group of 18.5–23.9 kg/m^2^ and the group of <18.5, 24.0–27.9, or ≥28.0 kg/m^2^, Table S3: Mean differences of BMD and HSA indices between the years of menstruation group of ≤31 years and the group of 32–36, 37–41, or ≥42 years.

## Abbreviations

BMI: Body mass index
BMD: Bone mineral density
HSA: Hip Structure Analysis
ROI: Regions of interest
NN: Narrow neck
IT: Intertrochanter
FS: Femoral shaft
CSA: Cross-sectional area
OD: Outer diameter
CT: Cortical thickness
SM: Section modulus
BR: Buckling ratio
